# Graphene-Based Semiconductor Heterostructures for Photodetectors

**DOI:** 10.3390/mi9070350

**Published:** 2018-07-13

**Authors:** Dong Hee Shin, Suk-Ho Choi

**Affiliations:** Department of Applied Physics and Institute of Natural Sciences, Kyung Hee University, Yongin 17104, Korea; sdh0105@hanmail.net

**Keywords:** graphene, photodetector, transparent conductive electrode, hybrid heterostructure

## Abstract

Graphene transparent conductive electrodes are highly attractive for photodetector (PD) applications due to their excellent electrical and optical properties. The emergence of graphene/semiconductor hybrid heterostructures provides a platform useful for fabricating high-performance optoelectronic devices, thereby overcoming the inherent limitations of graphene. Here, we review the studies of PDs based on graphene/semiconductor hybrid heterostructures, including device physics/design, performance, and process technologies for the optimization of PDs. In the last section, existing technologies and future challenges for PD applications of graphene/semiconductor hybrid heterostructures are discussed.

## 1. Introduction

A photodetector (PD) is a device for detecting light, the underlying mechanism is conversion of light to an electrical signal called a photocurrent (PC). PDs are used in a wide range of academic and industrial fields, including image sensing, optical communications, environmental monitoring, and chemical/biological sensing [1,2,3]. In PDs, usually made of inorganic or organic semiconducting materials, incident photons are absorbed and subsequently electron-hole pairs are generated, thereby producing PC. PDs are classified mainly as photodiodes, photoconductors, and phototransistors. Despite increasing reliability of the fabrication processes, their high cost/complexity and the large driving voltage of PDs have limited the diffusibility, compatibility, and versatility of PDs for broad applications and new technologies. In order to solve these problems, many studies have focused on how to develop simple processes for high-performance PDs, especially by using transparent conductive electrodes (TCEs) such as metal nanowires (NWs) [4,5], conducting polymers [6], carbon nanotubes [7,8], and graphene instead of metal or transparent conducting oxide electrodes. Among them, graphene is recognized as one of the next-generation TCEs, as explained below. Firstly, the extremely high carrier mobility of graphene enables ultrafast conversion of photons or plasmons to electrical signal, which is highly desirable for high-speed photodetection [9,10]. Secondly, the tunable electrical and optical properties of graphene, such as carrier densities, band alignments, and polarities, via chemical or electrostatic doping offer great flexibility for optimizing the performance of graphene-based optoelectronic devices [11,12]. 

A wide variety of optoelectronic devices based on graphene are still being studied, and some of them have already reached a level of competitiveness comparable to conventional semiconductor devices [13,14]. However, single-layer graphene has low light absorbance (only 2.3%) in the ultraviolet (UV) to near infrared (NIR) region and short light-matter interaction length, unfavorable for light harvesting applications [15,16]. In addition, the ultra-short lifetime of excitons in pure graphene resulting from its gapless nature also leads to fast carrier recombination, which limits the efficient production of PC or photovoltage [17,18]. Graphene itself can act as an absorber in ultrafast and ultra-broadband detectors, but the sensitivity of graphene-based detectors is relatively low, not suitable for most practical applications.

The emergence of the graphene/SiC interface in 2009 [19] as the prototype of graphene/semiconductor heterostructure has attracted much attention due to the expected synergistic properties of the two materials. Afterwards, the rapid development of graphene transfer techniques has led to a wide variety of unique designs for the functionality of the device geometry based on graphene/semiconductor hybrid heterostructures. Unlike other detector structures, the best feature of graphene/semiconductor junctions is the adjustable Schottky barrier height, useful not only for understanding the interface transport mechanism, but also for adjusting the device functionalities. Recent studies have shown that graphene/semiconductor interfaces/heterojunctions are efficient for generation, separation, and transmission of photocarriers, thereby exhibiting new features in their optoelectronic applications [20,21,22,23]. A broad review of recent developments in this area will not only offer new insight into light–matter interactions, but it will also help inspire future nontraditional next-generation detector designs.

Chemical vapor deposition (CVD)-grown graphene was first employed in graphene/Si heterojunctions, resulting in 65% external quantum efficiency (EQE) [24], which served as the prototype for the subsequent intensive studies on the graphene TCEs-based various kinds of semiconductor hybrid heterojunctions for high-efficiency PDs [24,25,26,27,28,29,30,31,32,33,34,35,36,37,38,39,40,41,42,43,44,45,46,47,48,49,50,51,52,53,54,55,56,57,58,59,60,61,62,63,64,65,66,67,68,69,70,71,72,73,74,75,76,77,78,79,80,81,82,83,84,85,86,87,88,89,90,91,92,93,94,95,96,97,98,99,100]. Here, we review recent progress in the development of graphene TCEs-based semiconductor hybrid heterojunction PDs. Despite the frequent use of mechanical cleavage and chemical exfoliation for graphene, we focus on CVD-grown graphene for PDs, most popular for practical device applications at the moment. First, we briefly explain key figure-of-merit parameters that are used to characterize PDs. In the main section, we discuss the issues related to physics/design, processing technologies, and performance of PDs fabricated by using various kinds of materials. Finally, we summarize the technologies reported so far and the significance/outlook for guiding continuing development of the graphene-TCEs-based PDs.

## 2. Operation Mechanisms 

Graphene/semiconductor hybrid heterostructures can be formed by directly combining graphene with one-, two-, and three-dimensional semiconductors through relatively simple manufacturing processes. When graphene and a semiconductor material form a junction, charge transfer occurs until the Fermi energy levels of the two materials coincide because their work functions mismatch. Meanwhile, the free charge region inside the semiconductor near the interface is depleted by the charge transfer, resulting in the formation of the built-in electric field. In case of graphene/n-type semiconductor junction, the immobile positive charge formed by the depletion of electrons bends the semiconductor band near the interface upward. More details are given in previous reports [101,102]. In the equilibrium state where the Fermi energy levels are aligned, the discontinuity of the allowed energy states of the two materials creates an energy barrier (Φ_B_) to prevent the electrons from flowing from the semiconductor to the graphene. Depending on the energy of the radiation (*E*) = *hν* (*h* = Planck’s constant and *ν* = photon frequency), PC is generated based on two main mechanisms. (1) When Φ_B_ < *hν* < semiconductor band gap (*E_g_*), the electrons are excited from the graphene and are injected into the semiconductor; (2) When *hν* > *E_g_*, the electron-hole pairs are generated in the depletion layer of the semiconductor. The PDs are operated based on the photovoltaic or photogating effect depending on their device configurations.

### 2.1. Photovoltaic Effect

The PD can be operated in a photovoltaic mode (=zero bias). The heterostructures generally exhibit rectifying behaviors, i.e., nonlinear current density-voltage (*J-V*) characteristics under dark. A PD operating in this mode is named as a photodiode, whose linearity, detectivity, and sensitivity are maximized due to the lowest dark current. In contrast, the responsivity (*R*) is usually low due to the lack of internal gain. The PD can be also operated in reverse bias (photoconductive mode), where the built-in external electromagnetic field enhances the separation efficiency of the electron-hole pairs, thereby reducing the response time due to the shortening of the passage time and the decrease of the diode capacity. The photovoltaic mode is more suitable for precise photodetection with the photoconductive mode being better suited for high-speed applications.

### 2.2. Photogating Effect

Photogating effects occur in the heterostructures-based phototransistors. The operating mechanism of the phototransistors is as follows. Once electron-hole pairs are generated in semiconductors, one type of carrier is transferred to the graphene, and the opposite-type carrier is trapped in the semiconductor. The trapped charges act as a local gate, thereby effectively modulating the Fermi level of graphene through capacitive coupling. This induces more carriers and consequently modulates the electrical conductance. In addition, the transferred free carriers circulate many times in the graphene channel within their lifetime, which contributes to higher photoconduction gain. The gain is expressed as gain = lifetime/transit, where transit is the transit time of the photogenerated carriers through the graphene channel. Shorter transit time and longer lifetime should be obtained to achieve higher gain. Nevertheless, the lifetime of the photocarrier is determined by the response time, which is directly related to the carrier recombination process. Thus, a high gain will extend the response time. A phototransistor can show much higher *R* than a photodiode, but the response speed of the former is slower than that of the latter. Due to the effective gate field of the trapped carriers, the photogate effect generally appears as a horizontal shift of the source-drain current-gate voltage (*I_SD_-V_g_*) curve of the graphene transistor upon illumination. In addition, the direction of the curve shift represents the polarity of the captured carriers, and the photogate effect can induce positive or negative PC by *V_g_*_._

## 3. Figure of Merits for Characterizing Photodetectors

### 3.1. Responsivity (R)

*R* is defined as the ratio of PC to incident light power, which indicates how efficiently a PD responds to an optical signal, as expressed by the equation *R* = *I_ph_/L_light_*, in a unit of A/W, where *I_ph_* is the PC and *L_light_* is the incident-light power. *R* is proportional to EQE of a PD, meaning the conversion rate from photons to electrons/holes. Therefore, EQE is related to *R* based on the following equation:

*R* = *EQE* × *λq*/*hc* (1)

where *λ* is the incident-light wavelength, *q* is the absolute value of electron charge, *h* is the Planck constant, and *c* is the speed of light.

### 3.2. Detectivity (D*)

D* is the weakest level of light which can be detected by the device, and is determined by *R* and noise of a PD. D* is given by D* = (A·∆f)^1/2^ *× R/i_n_*. Where *A* is the effective area of a PD, *∆f* is the electrical bandwidth, and *i_n_* is the noise current. The performance of D* is limited by three kinds of noise such as dark current (DC), Johnson noise, and thermal fluctuation noise [103]. By assuming the shot noise as the dominant contribution, D* is expressed by the formula: D* = *R/(2qJ_d_)^1/2^* where *J_d_* is the DC density.

### 3.3. Noise Equivalent Power (NEP)

Noise should be kept as low as possible because it determines minimum detectable signal. There are several kinds of noises such as thermal noise, shot noise, and flicker noise, composing the total noise. In this context, NEP is an associated figure of merit, and is defined as the optical power that yields a single signal-to-noise ratio for a given bandwidth, in a unit of W*·*Hz^−1/2^. NEP also represents minimum detectable power and is calculated by the following equation.

*NEP* = *i_n_*/*R* = (*A*·*∆f*)^1/2^/D* (2)

### 3.4. Linear Dynamic Range (LDR)

LDR describes an illumination intensity range within which the current response of a PD scales linearly with the light intensity. LDR is given by the equation: LDR = 20 log(J_ph_*/J_d_) where J_ph_* is the PC density measured at a light intensity of 1 mW·cm^−2^. For practical applications, it is desirable to have a large LDR for a PD capable of detecting both weak and strong light. 

### 3.5. Response Speed

The response of a PD to an optical signal is characterized by the rise/fall times, defined as the times at 10/90% of the maximum PC, respectively, which are strongly related to the charge transport/collection and the bandwidth of the photoresponse.

In the following sections, the PD characteristics are detailed for various kinds of graphene TCE/semiconductor heterostructures, whose major figure of merit parameters are summarized in Table 1.

## 4. Graphene/Silicon

### 4.1. Doping of Graphene

The built-in electric potential at the graphene/Si interface is determined by the difference between the work functions of graphene and Si when no bias is applied. Pristine graphene forms a low Schottky barrier with Si due to the relatively-small work function (4.5 eV), and the pristine graphene/Si junction exhibits a large series resistance due to the relatively large sheet resistance of pristine graphene. These issues should be overcome to enhance the performance of the graphene/Si PDs. Chemical doping is a very efficient approach for increasing the work function and conductivity of graphene, resulting in the increase of the Schottky barrier and the reduction of the series resistance. The electrons and holes are then less recombined, thereby improving the performance of the PDs. 

### 4.2. Graphene/Silicon Wafer

**Figure 1:**
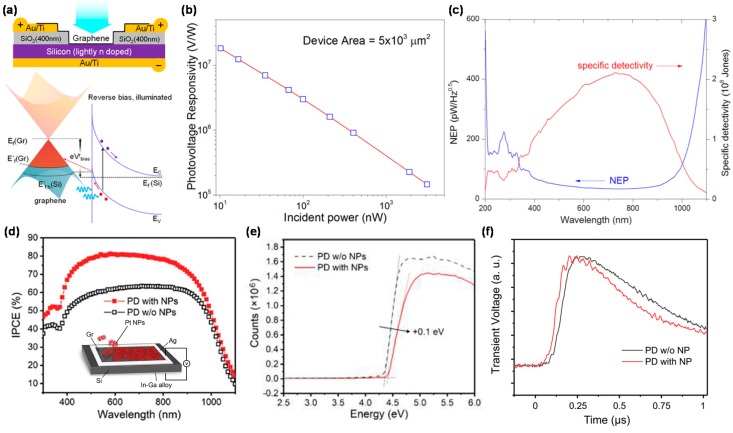
(**a**) Schematic diagram and band structure of a typical monolayer graphene/Si heterojunction device. Application of a reverse bias raises *E_f_* (graphene: Gr) and opens up a large number of accessible states that can be occupied by photoexcited holes injected from Si under illumination; (**b**) Variation of the voltage responsivity as a function of incident power, obtained under the open-circuit condition. At the lowest powers, the voltage responsivity exceeds 10^7^ V/W; (**c**) Spectral dependence of the NEP and specific detectivity (D*) in the photocurrent mode. Reproduced with permission for Figure 1a–c from 2013 Nano Letter [24]; (**d**) Incident photon conversion efficiency (IPCE) spectra of graphene-Si PDs with and without fractal Pt NPs. The inset shows schematic diagram of a Si-graphene PD with fractal Pt NPs; (**e**) Ultraviolet photoelectron spectroscopy data of graphene-Si PDs with and without fractal Pt NPs, showing the effect of physical doping on the Fermi level; (**f**) Transient photovoltage characteristics of graphene-Si PDs with and without fractal Pt NPs under 532 nm pulse laser. Reproduced with permission for Figure 1d–f from 2017 Advanced Optical Materials [29].

In the last decade, graphene/Si heterostructures have been studied for optical detection based on a powerful photovoltaic effect. In these structures, light absorption takes place in Si whilst graphene acts as an electrode for efficient carrier transport and collection. Early studies have shown that the graphene/Si Schottky junctions, introduced in 2013 (Figure 1a) [24], were very sensitive to the illumination in broadband wavelengths [24,25,26], thereby showing *R* and NEP greater than 10^7^ V/W and 1 pW·Hz^−1/2^, respectively, in optical voltage mode, as shown in Figure 1b,c. The Fermi level of graphene was controlled by 1-pyrenecarboxylic acid doping, allowing smooth transport/collection of high-density holes photogenerated in Si under illumination, resulting in 0.435A/W *R*, 65% EQE, and 7.69 × 10^9^ cm·Hz^1/2^·W^−1^ Jones D*. 

The gapless and semi-metallic properties of graphene are very promising for its application for broadband photodetection from microwave to UV wavelengths. Graphene/Si Schottky PDs are also very efficient in fast carrier separation due to the presence of the built-in electric field without suffering from a sharp reduction in the lifetime of photocarriers under 1550 nm illumination [25], thereby showing an *R* of 0.28 A/W, corresponding to an internal quantum efficiency of 10%, much higher than a typical Schottky junction (~1%). Usually, the R in the UV region is at least 1–2 times lower than that in the visible-near infrared (NIR) region due to the weak light absorption and relatively short carrier lifetime of the graphene. TiO_2_ [27] and Al_2_O_3_ anti-reflection layers [28] have been employed to enhance the photoresponse in the UV region. Recently, graphene/Si Schottky structure with an Al_2_O_3_ antireflective layer has been fabricated for UV photodetection, resulting in 0.2 A/W *R*, 5 ns response time, 1.6 × 10^13^ cm·Hz^1/2^/W D* in wavelength range from 200 to 400 nm, comparable to those of state-of-the-art Si, GaN, and SiC Schottky PDs. 

Unlike traditional transparent electrodes, such as indium tin oxide or ultrathin metals, unique absorption properties of graphene in the UV range contribute to increasing the lifetime of hot electrons, thereby leading to large PC. Recently, a variety of graphene/Si-based structures have been proposed to improve the device performance. A new approach of integrating graphene/Si PD with two-dimensional (2D) Pt nanoparticles (NPs) has proven to enhance the *R* and response time of the PD at the same time, as shown in Figure 1d [29]. Furthermore, the work function of graphene is increased by the physical doping with Pt NPs, thereby heightening the barrier of the Schottky junction, resulting in faster response and higher sensitivity, as shown in Figure 1e,f. Molybdenum oxide (MoO_3_) has been widely used for improving the performance of the PD in organic electronics due to its high work function [104,105] and dramatic surface-transfer hole-doping effect for a variety of 2D materials such as graphene and MoS_2_. The functionalization of the MoO_3_ layer on graphene led to a significant performance enhancement of the graphene/Si PDs [30], based on the surface-transfer doping effect, thereby reducing the series resistance of the graphene/Si Schottky barrier, resulting in 5.4 × 10^12^ cm·Hz^1/2^/W D*. However, the interface between graphene and Si normally contains relatively-high density of surface states, resulting in large leakage current, which can limit the overall D* of the graphene/Si PD [24,25]. Recently, the DC of the graphene/Si junction was reduced by two orders at 0 bias by employing a thin interface thin oxide layer [31]. The graphene/thin SiO_2_/Si PD showed 0.0078 pW·Hz^−1/2^ NEP at room temperature, resulting in D* up to 4.08 × 10^13^ cm·Hz^1/2^/W. 

In addition, graphene/Si Schottky diodes were fabricated in parallel with graphene/SiO_2_/Si capacitors for high-performance optical detection [32], thereby showing R/D* of 3 A·W^−1^/3.5 × 10^12^ cm·Hz^1/2^·W^−1^ in the visible range. Here, the PC exceeded the forward current because the photogenerated minority carriers accumulated in the Si/SiO_2_ interface diffused to the graphene/Si junction. According to another approach, the PC in graphene-based hybrid photodiodes was not only very efficiently generated in the graphene/Si Schottky barrier region but also in the adjacent graphene/SiO_2_/Si region due to the reverse bias voltage [33]. The PC in the graphene/SiO_2_/Si region rose sharply by about one order of magnitude at voltages exceeding a certain threshold bias, regardless of the incident laser power. In Si under SiO_2_, the inversion layer not only provided a low resistance path for minority charge transport, but also acted as a passivation layer on the surface states of SiO_2_ to effectively collect the charge carriers, resulting in improved PC. These results provide guidelines for establishing the fundamental mechanism of PC generation in graphene-based hybrid optoelectronic devices and for designing hybrid photodetectors based on combinations of two- and three-dimensional materials. The PD was also fabricated by directly growing graphene nanowalls on a Si substrate instead of using graphene layers CVD-grown via a metal catalyst [27]. Resulting D* was 5.88 × 10^13^ cm·Hz^1/2^/W, higher than ever reported in the planar graphene/Si based PDs, thanks to the ultra-low current noise of 3.18 fA·Hz^1/2^, which can be attributed to the excellent junction quality of 0.69 eV barrier height and 1.18 ideal factor. Other principal PD parameters were 10^7^ PC/DC (*I_light_/I_dark_*) ratio, 40 μs response time, 8.5 kHz cutoff frequency, and 0.52 A/W *R*. 

### 4.3. Graphene/One- and Two-Dimensional Silicon

**Figure 2:**
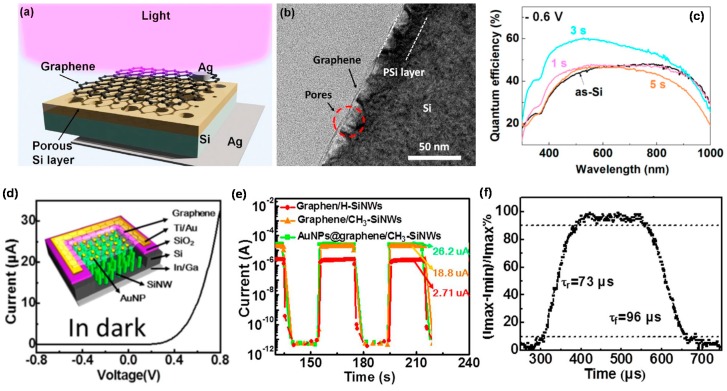
(**a**) Schematic diagram of graphene/PSi/n-Si PD with silver top and bottom electrodes under light illumination; (**b**) Cross-sectional transmission electron microscopy (TEM) image of graphene/PSi junction; (**c**) Quantum efficiencies of as-Si, 1, 3, and 5 s PDs (Here, 1, 3, and 5 s mean the deposition time (*t_d_*) of Ag nanoparticles. That is, the pore on the surface of the PSi increases as the time increases, Reproduced with permission for Figure 2a–c from 2014 ACS Applied Materials & Interfaces [35]; (**d**) Current-voltage curves of the Au NPs-graphene/SiNWs array PD. The inset shows the device structure; (**e**) Photoresponse of three representative devices under 850 nm light illumination at *V* = 0; (**f**) A single normalized cycle measured at 2200 Hz for estimating both rise time (*t_r_*) and fall time (*t_f_*). Reproduced with permission for Figure 2d–f from 2014 Scientific Reports [36].

Two-dimensional Si nanostructures such as porous Si (PSi) and Si NWs have high ratio of surface to volume/light absorption, very useful for their application in graphene-based PDs. Figure 2a shows a schematic diagram of a graphene/PSi Schottky diode. Graphene/PSi Schottky diodes have been successfully fabricated to show excellent PD performance including 60% EQE and 3μs response time, relatively higher than the graphene/Si wafer PDs in the near-UV wavelength range, as shown in Figure 2c [35]. The performance of the graphene/PSi PDs can be further improved by optimizing the device structure and the pore depth of PSi. Alternatively, the NIR PDs fabricated based on a structure of graphene/Si NW array decorated with Au NPs [36] showed high performance of 10^6^ *I_light_/I_dark_* ratio and high response speed (rise/fall time of 73/96 μs), better than those of pure graphene/Si NWs heterojunction [106], as shown in Figure 2c,d. These high PD parameters possibly resulted from the enhanced optical absorption due to the strong optical trapping effect of Si NW arrays and the surface plasmon coupling by Au NPs.

### 4.4. Graphene/Zero-Dimensional Silicon

**Figure 3:**
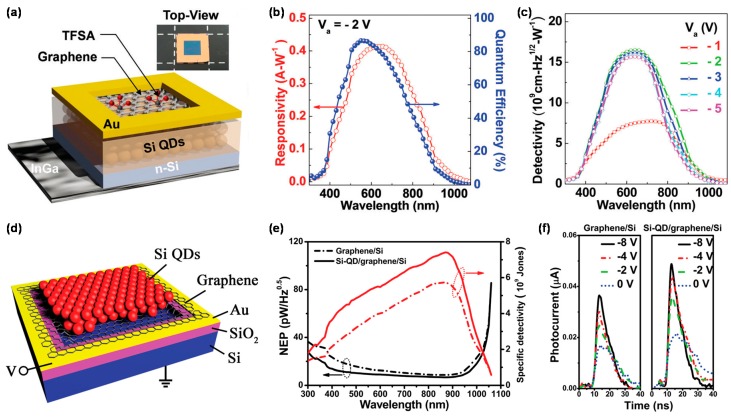
(**a**) Schematic of a typical TFSA-doped graphene/SQDs: SiO_2_ MLs/n-type Si wafer PD with Au and InGa as the top and bottom electrodes, respectively; (**b**) Spectral responsivity and quantum efficiency at a bias of −2 V; (**c**) Spectral detectivity under various bias voltages. Here, the doping concentration is 20 mM. Reproduced with permission for Figure 3a–c from 2017 Journal of Materials Chemistry C [39]; (**d**) Schematic diagram of a typical Si-QDs/graphene/Si PD; (**e**) Spectral dependence of the NEP and corresponding specific detectivity of the graphene/Si and Si-QDs/graphene/Si PDs. The minimum NEP and the maximum D* of the Si-QDs/graphene/Si PD are 6.7 pW·Hz^−1/2^ and 7.4 × 10^9^ Jones, respectively, at *λ* ≈ 877 nm; (**f**) Time-dependent PC of both the graphene/Si and Si-QDs/graphene/Si PDs under the bias voltages of 0, −2, −4, and −8 V. Reproduced with permission for Figure 3d–f from 2016 Advanced Materials [40].

Si is widely used as a broadband PD, but is limited in its use for wider applications due to the inherent indirect bandgap nature. Especially, graphene/Si-based PD exhibits fast photoresponse in the UV and NIR regions, but not in the visible region. One way to overcome this drawback is to form Si quantum dots (SQDs) [107,108]. SQDs within a SiO_2_ matrix allow for large light absorption in the visible range based on the quantum confinement effect [37,38]. Recently, AuCl_3_-doped graphene/SQDs: SiO_2_ multilayers (MLs)/Si PDs were successfully fabricated to show 0.35 A/W *R* and 10^9^ cm·Hz^1/2^/W D* near the wavelength of 600 nm, indicating considerable performance improvement in the visible region due to the strong photoresponse of SQDs [37,38]. The novel DC/PC behaviors are dominated by the tunneling of the charge carriers, depending on the SQD size and AuCl_3_ doping concentration (*n_D_*). However, the performance of the PD is limited by the surface of the AuCl_3_-doped graphene on which light is first irradiated due to large reduction in transmittance by the Au NPs formed by the doping. Recently, long-term stabilities have been remarkably enhanced by employing bis(trifluoromethanesulfonyl)-amide (TFSA) with low resistance and high transparency as a dopant for graphene, as shown in Figure 3a [39]. Figure 3b,c shows spectral *R* and D* of the TFSA-doped graphene/SQD: SiO_2_ MLs/Si PDs with 0.413 A/W and 10^10^ Hz^1/2^/W at the peak wavelength of 630 nm under low voltage (−2 V), respectively, significantly better than the AuCl_3_-doped graphene PDs, indicating minimization of the optical loss by the use of TFSA. On the other hand, graphene/Si Schottky junction PDs with SQDs on top of the graphene, as show in Figure 3d [40], showed excellent performances of 6.7 pW·Hz^−1/2^ NEP, 7.4 × 10^9^ cm·Hz^1/2^/W D*, and response time ≤ 25 ns, possibly resulting from significant reduction of the optical reflections, contributed by the SQDs, as shown in Figure 3e,f. In another approach, high-sensitivity NIR detection of SQDs/graphene/Si hybrid phototransistors was demonstrated based on the plasmonic effect of SQDs doped with boron [41]. The local surface plasmon resonance of B-doped SQDs improves the NIR absorption of graphene, resulting in excellent light output characteristics in the UV to NIR region. 

## 5. Graphene/III-V Semiconductors

### 5.1.Graphene/GaN

**Figure 4:**
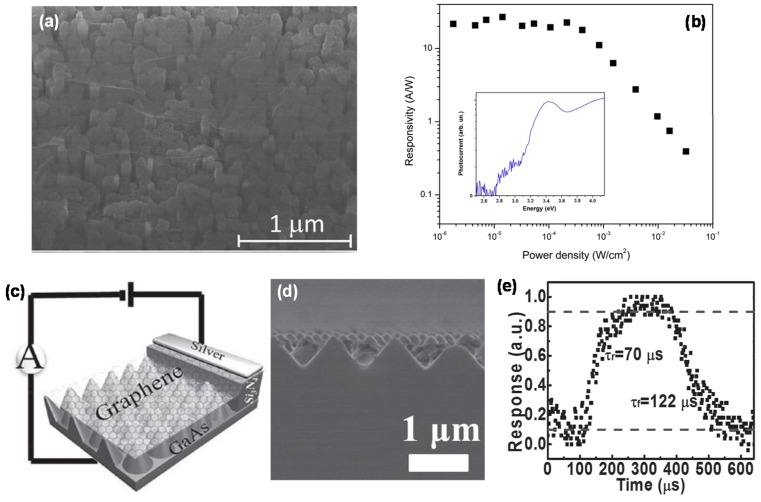
(**a**) Scanning electron microscopy (SEM) image of a graphene film on GaN NWs; (**b**) Responsivity for various incident power densities. The inset shows the photocurrent spectrum of the PD. Reproduced with permission for Figure 4a,b from 2013 Applied Physics Letters [43]; (**c**) Schematic illustration of the graphene/GaAs nanocone array device; (**d**) A typical cross-sectional SEM image of the *n*-GaAs nanocones array after etching; (**e**) Single normalized cycle at 50 Hz to find both rise time (*τ_r_*) and fall time (*τ_f_*). Reproduced with permission for Figure 4c–e from 2014 Advanced Functional Materials [44].

GaN is a wide direct bandgap semiconductor with excellent optical/electrical properties and high chemical stabilities at high temperature, and has been employed in various PD applications. Graphene/GaN heterostructures are useful for light detection in the UV/visible region based on Schottky diode junction [42,43]. Graphene-TCE based GaN Schottky diode for UV detectors [42] has shown excellent performance of high *I_light_/I_dark_* ratio (10^5^) under UV illumination. In another approach, UV PDs were successfully fabricated by transferring large-area graphene TCE on vertically-standing GaN NW array as a light absorbing media, as shown in Figure 4a [43]. Figure 4b shows extremely-high *R* of 25 A/W at 357 nm. These results represent a potential application of the graphene/GaN junctions in various optoelectronic devices operating in the UV region.

### 5.2. Graphene/GaAs

GaAs has long been considered as an ideal candidate material for NIR PD applications due to its striking electrical/optical properties such as high carrier mobility, direct bandgap of ~1.4 eV, and large optical absorption coefficient. As shown in Figure 4c,d, graphene TCE was applied for GaAs nanocone array-based NIR light detection [44], resulting in reproducible PD performance of 10^4^ *I_light_/I_dark_* ratio, 1.73 mA/W *R*, and 10^11^ cm·Hz^1/2^/W under 850 nm illumination. The PD also showed fast response speed with rise/fall times of 72/122 μs, as shown in Figure 4e. These results are believed to originate from the GaAs nanocone arrays, which can efficiently trap incident NIR light, consistent with previous report [45]. Recently, AlO_x_ thin layer was employed for optimizing *R* of the graphene/GaAs NIR PD. Here, the AlO_x_ serves not only as a surface protective layer for suppressing the carrier recombination, but also as a barrier for reducing the current leakage [46], thereby leading to significant increase/decrease in PC/DC, respectively. With the use of the AlO_x_ layer, the *R* and specific D* increased by 4 times to 5 mA/W and 2.8 × 10^11^ cm·Hz^1/2^/W, respectively, whilst rise/fall times sharply decreased to 320/380 ns.

## 6. Graphene/Metal Oxides

### 6.1. Graphene/ZnO

**Figure 5:**
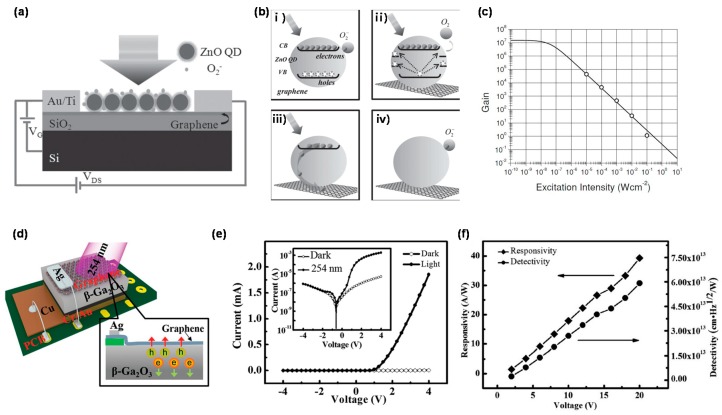
(**a**) Schematics of the graphene device coated with ZnO QDs; (**b**) The mechanism of the oxygen-assisted charge transfer process. (i) With irradiation of photon energy larger than the bandgap of the QDs, electron-hole pairs are generated; (ii) The holes are trapped at the surface states, leaving behind the unpaired electrons. The trapped holes will discharge the oxygen ions on the surface, and the oxygen molecules are desorbed; (iii) The electrons will transfer from the QDs to the graphene layer; (iv) With laser turned off, no more electron-hole pairs are generated. The oxygen molecules will re-adsorb on QDs and capture the remaining electrons to form oxygen ions on the surface; (**c**) Gain as a function of excitation intensity. Reproduced with permission for Figure 5a–c from 2013 Small [49]; (**d**) Schematic diagram of the MLG/β-Ga_2_O_3_ PD; (**e**) Current-voltage characteristics of the device under dark and 254 nm-light irradiation. The inset shows the current-voltage curves on a logarithmic scale (**f**) Responsivity and detectivity of the multi-layer graphene-Ga_2_O_3_ heterojunction PD as a function of different bias voltages. Reproduced with permission for Figure 5d–f from 2016 Advanced Materials [69].

ZnO has received considerable attention over the past two decades due to its unique properties such as direct bandgap of 3.2 eV, large exciton binding energy of ~60 meV, high transmittance (>80%) in the wavelength range of 400–800 nm, and wide-variable conductivity in the metallic to insulating range [109,110]. To date, graphene/ZnO heterostructures have been extensively studied for PD application. Recently, a seedless solution process has been developed for controllable growth of crystalline ZnO micro-/nano-wire arrays on graphene for UV PDs [47,48,49,50,51,52], showing high response of 1.62 A/W, better than the sensors made of epitaxial-ZnO [111] or ZnO-nanoparticles [112]. In another approach, novel ZnO-QDs-based heterostructures were combined with graphene layers for PDs to show excellent visible-blind UV photoresponse [48], resulting from the reduction of the DC, caused by the formation of the potential barriers between adjacent ZnO QDs and between graphene and ZnO QDs. It has also been reported that phototransistors composed of ZnO nanostructure as light harvesting medium and graphene as conducting channel [49,50,51,52,53,54,55,56]. Figure 5a,b shows the effective charge transfer between ZnO QDs and graphene [49]. Under UV irradiation, the holes separated from the electrons under photoexcitation are captured by the surface states to discharge the absorbed oxygen ions on the surface of the ZnO QDs. The unpaired electrons with a relatively long lifetime move to the graphene layer and then transport in the graphene channel, leading to modulation of the transport characteristics of graphene. When UV light is turned off, the surrounding oxygen molecules are re-adsorbed on the QD surface, thereby trapping electrons and lowering the Fermi energy level, resulting in the recovery of the transport characteristics of the graphene. This photogeneration effect could lead to high photoconductive gain of 10^7^ through further device optimization, as shown in Figure 5c.

In recent years, ZnO NW or nanorod (NR) arrays have been combined with various graphene sheets on rigid or flexible substrates to form UV phototransistors [50,51,52,53,54]. ZnO NR array was grown on the channel of the graphene field-effect transistor (FET) by the hydrothermal method to successfully fabricate the UV PDs [50], showing high sensitivity/wavelength selectivity, but still quite a slow response/recovery under UV illumination at low power. In other studies, UV phototransistors were fabricated with organic self-assembled monolayer (SAM), sandwiched between inorganic ZnO-QDs-decorated graphene channel and conventional SiO_2_/Si substrate [55]. Surprisingly, the mobility of the chemically-deposited graphene channel is higher on the SAM than on the SiO_2_, thereby greatly reducing the time of the electron transfer in the channel. As a result, the photogenerated electrons transferred from the QD to the graphene are recycled several times before recombination, resulting in the increase of the photoconductivity gain. The phototransistors exhibited ~10^8^ A/W *R* corresponding to ~3 × 10^9^ gain, ~10^3^ UV/visible rejection ratio, and ~5.1 × 10^13^ cm·Hz^1/2^/W specific D*. More recently, phototransistors consisting of graphene FET and ZnO QDs have been fabricated based on surface active process [56]. Resulting D* increased up to 7.5 × 10^14^ Jones due to the efficient transfer of charge carriers from ZnO QDs to graphene. These results suggest how important the van der Waals interface is for the efficient optoelectronic processes in heterostructure PDs. On the other hand, in graphene/ZnO heterostructures photodiodes [57,58,59,60,61,62,63,64,65,66,67], the photogenerated electron/hole pairs were found to be separated by the built-in potential even at zero bias, thereby potentially reducing the DC, resulting in the increase of specific D* [61,64,66]. The response of the photodiode was shown to be much faster than that of the phototransistor-type PD by further increasing the built-in electric field under moderate reverse bias [57,59,60,62,63]. In addition, large internal current gain could also be obtained due to the impact ionization of the photocarriers with sufficient energy [16]. 

A Schottky-junction-type UV PD fabricated by depositing graphene layer on ZnO NR array was shown to be highly sensitive to UV illumination with excellent operational stability and reproducibility, resulting in 113 A/W *R* and 385 gains at −1 V [57]. The response speed was remarkably fast (rise/fall time: 0.7/3.6 ms) due to the strong light trapping effect by high-crystallinity ZnO NR array. The PD could also monitor the pulse light at high frequencies of 2250 Hz. The PD performance was further improved by employing charge tunneling/blocking interlayer [58] and by modulating the Schottky barrier through back gating [59] or external deformation [60,62]. The R was also enhanced up to 1350 A/W at ~5 V by inserting a hexagonal boron nitride (h-BN) layer into the graphene/ZnO Schottky junction [58]. The h-BN interface layer reduced the DC by suppressing the electron transfer from graphene to ZnO, and suppressed the recombination of carriers, resulting in the increase of the PC, by holding photogenerated electrons in ZnO. 

Recently, the effect of strain modulation on PD performance was studied for a flexible graphene/ZnO nanostructure [60,65]. The *R*, measured under an external strain of 5 to 30%, showed maximum 17 μA/W and 3.8 μA/W at a strain of 30% for UV and visible light, respectively [65]. The compressive strain causes ZnO to generate a negative piezoelectric potential, and the charge begins to accumulate near the conduction band, lowering the conduction band energy. When the conduction band energy is lowered, the Schottky barrier is increased and the sensitivity of the graphene/ZnO PD is improved due to an increase in the width of the depletion region, which is advantageous for the photogenerated electron-hole pair separation. The theoretical simulation results also showed that the piezo-potential was mainly distributed at the end of the ZnO nanostructure, advantageous for the effective modulation of the graphene/ZnO Schottky junction.

### 6.2. Graphenene/Other Metal Oxides

Like graphene/ZnO hybrid structures, other semiconductor metal oxides including ZrO_2_ [68], Ga_2_O_3_ [69,70] have also been integrated with graphene to form heterostructures for PD applications. ZrO_2_ is a promising material for deep-UV detection applications. Recently, in the ZrO_2_ QDs-graphene heterostructure, *R* was shown to be as high as 22 A/W at a deep-UV wavelength of 254 nm [68]. In the PD, the holes photo-excited in graphene can be circulated through the external circuit several times under bias voltage before recombining with the electrons photo-excited/collected in the ZrO_2_ layer due to the high mobility of graphene. This explains why *R* is so extremely high

As another approach, a deep UV photodiode was fabricated based on multilayer graphene/Ga_2_O_3_ junctions, as shown in Figure 5d [69], exhibiting a significant current increase at forward bias voltage during deep-UV illumination (254 nm), as shown in Figure 5e. Figure 5f shows the *R* and D* of 39.3 A/W and 5.92 × 10^13^ Jones, respectively, comparable to, or even better than, Ga_2_O_3_ nanostructure-based PDs. The PD parameters were reproducible and stable even by repeated measurements under UV illumination, showed maxima at ~220 nm, but were almost blinded to light at wavelengths longer than 280 nm. Most recently, graphene/Ga_2_O_3_/graphene PD showed apparent rectifying characteristics and excellent solar-blind UV photoresponse [70] with fast rise/fall times of 0.96/0.81 s and *I_light_/I_dark_* ratio up to 82.88 under 254 nm illumination. 

## 7. Graphene/Organic Semiconductors

Organic semiconductors including small molecules and polymers are very attractive in practical view due to their simple solution production processes at low temperatures [113], low cost, and light weight [114,115]. Graphene/P3HT hybrid heterostructures showed an *R* about 10 times larger on a piezoelectric substrate than on a SiO_2_ substrate [71]. The improved light response was attributed to the vertical field of the polarization of the piezoelectric substrate, which facilitates the spatial separation of the photo-generated electrons and holes, thereby enhancing the hole doping of graphene. 

**Figure 6:**
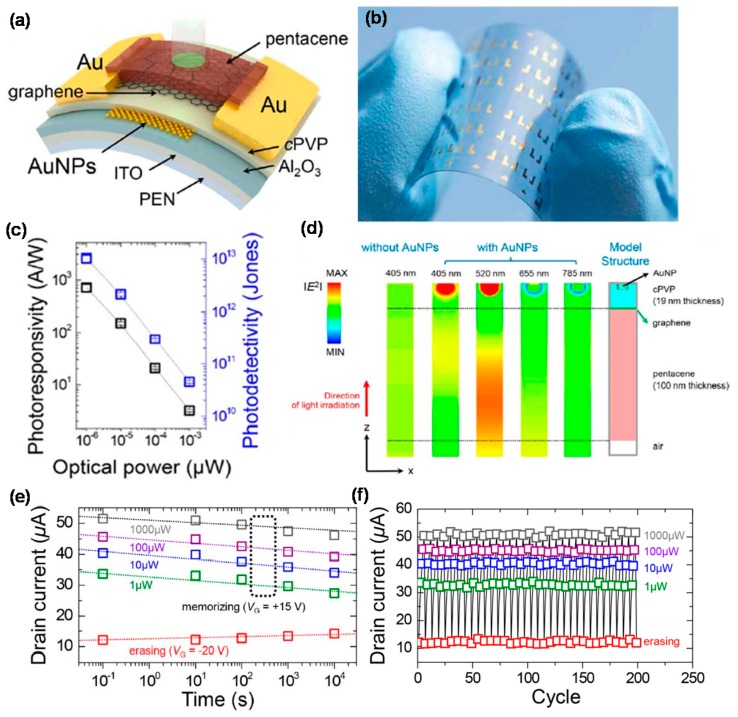
(**a**) Schematic diagram of the device structure comprising a graphene PD with a pentacene light absorption layer and an Au NP charge trapping layer. In the device, the ITO acts as a gate electrode and Au as source/drain electrode; (**b**) Photographic image of the graphene PD fabricated on a PEN substrate; (**c**) Photoresponsivity and photodetectivity vs. illumination power; (**d**) Two-dimensional spatial profile of *|E^2^|* of the hybrid PD with or without Au NPs; (**e**) Retention time and (**f**) cycling tests of the graphene PD with a memory functionality under various illumination powers. Reproduced with permission for Figure 6a–f from 2015 Nano Letters [72].

Figure 6a,b shows a typical graphene/pentacene heterostructure that serves as a multifunctional PD with nonvolatile memory (NVM) function for storing optical signals [72]. The PD exhibited excellent photoresponse at 400–800 nm with maximum *R* and specific D* being 700 A/W and of 10^13^ Jones, respectively, as shown in Figure 6c. Large hysteresis was observed in the transfer characteristics, in which the Dirac point voltage was shifted toward the positive gate voltage under dark and illumination. This hysteresis behavior was understood by the trapping/detrapping of the charge carriers in the Au NPs, resulting from the tunneling through the dielectric layer. The PD also showed excellent retention characteristics with retention times exceeding 10^4^ s and PC being invariant for more than 200 cycles (Figure 6e,f). It was also found that as the optical power increased, the PC increased stepwise, indicating multiple NVM. 

For a phototransistor-type PD structure, monolayer organic C8-BTBT crystals were prepared on graphene by van der Waals epitaxy [73], and was shown to exhibit the *R* larger than 10^4^ A/W. By employing multilayer C8-BTBT, the *R* was further enhanced to 4.76 × 10^5^ A/W. This outstanding photoresponse was attributed to the ultrahigh photoconductive gain and the efficient interfacial charge transfer efficiency due to the high quality of C8-BTBT layer and graphene/C8-BTBT interface. The response speed was ~25 ms for the monolayer PD, but increased to ~830 ms for the multilayer PD, dominated by interlayer hopping of trapped carriers within C8-BTBT crystals.

## 8. Graphene/Perovskites

**Figure 7:**
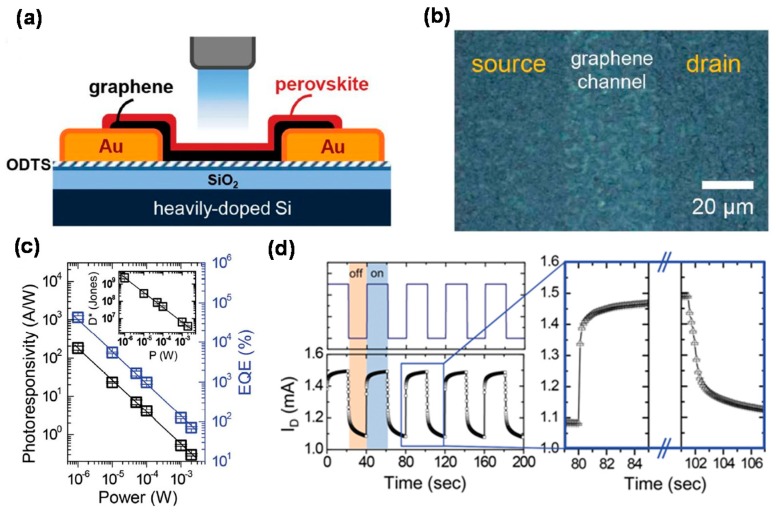
(**a**) Schematic diagram of the perovskite-graphene hybrid PD; (**b**) Optical microscopy image of the CH_3_NH_3_PbI_3_-graphene PD; (**c**) Photoresponsivity and EQE vs. illumination power. The inset shows the photodetectivity (D*) vs. illumination power; (**d**) On-off switching characteristics of the PD under dark and light illumination. By the pulsed laser illumination (left top), the temporal response of the photocurrent is shown (left bottom). Reproduced with permission for Figure 7a−d from 2014 Advanced Materials [74].

Perovskites have recently received strong attention as next-generation PD materials because of their high adsorption coefficient, tunable bandgap, small exciton binding energy, long carrier diffusion length, ambipolar charge transport, and high carrier mobility [116,117,118,119,120,121]. The perovskite material has a specific crystal structure as ABX_3_ formula. The larger *A* cations occupy cuboctahedral sites shared with twelve X anions, while smaller B cations stabilize at octahedral sites shared with six X anions. Organic-inorganic halide perovskite materials are commonly used in solar energy applications. The graphene/perovskite hybrid structure is very useful for taking advantage of the synergy effect based on the unique properties of graphene and perovskite materials. Figure 7 shows a typical early-stage phototransistor composed of graphene/CH_3_NH_3_PbI_3_ (MAPbI_3_) hybrid, exhibiting the *R* of several hundreds of A/W and relatively-rapid response time shorter than 1 s in the UV-visible range [74,75]. Especially, Figure 7d shows on-off switching behaviors of the perovskite-graphene PD with rise and fall times of 87 and 540 ms, respectively. These results show the robustness and reproducibility of the device.

**Figure 8:**
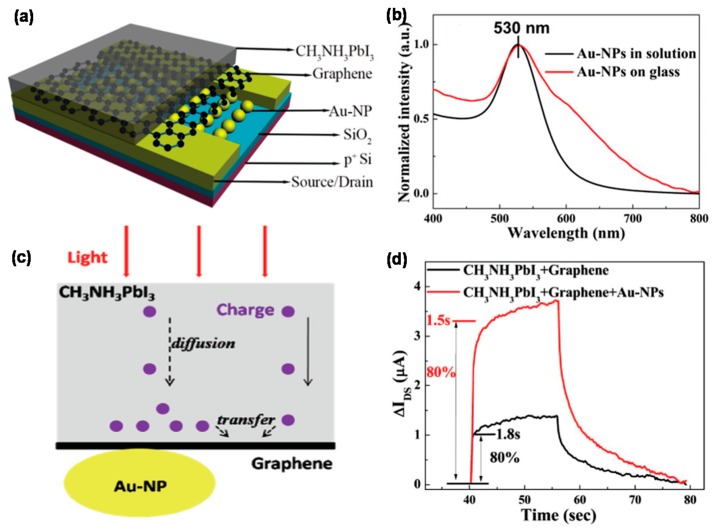
(**a**) Schematic of the CH_3_NH_3_PbI_3_-graphene-Au NP hybrid system device architecture (**b**) UV-Vis absorbance spectrum of Au-NPs on a 3-aminopropyltriethoxysilane-coated glass; (**c**) Schematic of generation, diffusion, and transfer of photo-induced carriers in the perovskite layer with and without the influence of gold NPs; (**d**) Response time study on a single representative light/dark cycle. Reproduced with permission for Figure 8a–d from 2016 Nanoscale [76].

It is currently difficult to compare the performances of the graphene/MAPbI_3_ hybrid phototransistors with those of graphene-based other semiconductor hybrid PDs operated based on similar mechanisms [21,22,122], but they are several times higher than those of pure graphene-based PDs [123]. The enhanced photoresponse performance can be attributed to the transfer of electrons from the graphene to the proximate perovskite layer to fill the empty states in the valence band of the perovskite layer, resulting in the reduction of the recombination of the photocarriers. The electron trapping process was demonstrated by the dramatic quenching of the PL intensity in the graphene/perovskite system. In another approach, the graphene/MAPbI_3_ stack was integrated with Au NPs having a plasmon resonance peak at ~530 nm, as shown in Figure 8, to fabricate the hybrid phototransistors showing higher *R* and faster response speed [76]. The higher *R* was attributed to the light-harvesting effect enhanced by the near-field of the perovskite, induced by the plasmonic coupling of Au NPs. Furthermore, the time for these carriers to diffuse into graphene is shorter than that for carriers generated far from the perovskite–graphene interface, thus leading to the faster operation in the PD by the plasmonic effect.

**Figure 9:**
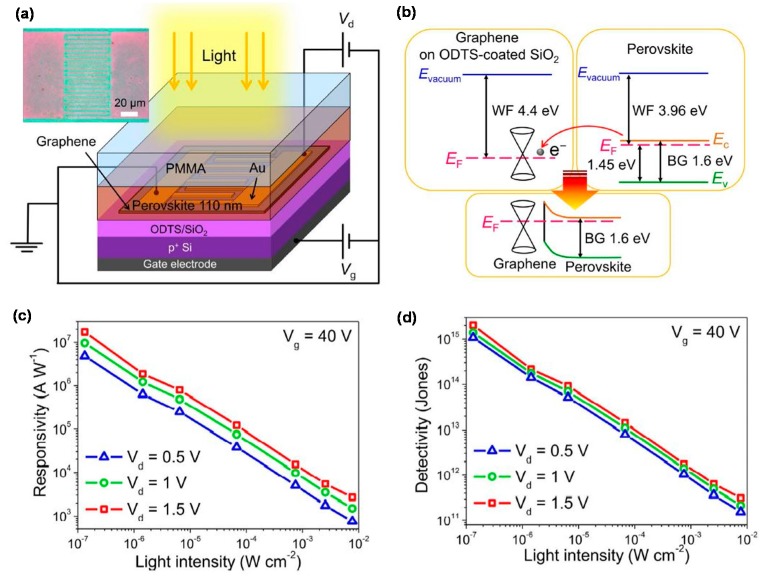
(**a**) Schematic illustration of the graphene–perovskite hybrid phototransistor. The inset shows the top-view optical microscopy image of the device; (**b**) Band diagrams of graphene and perovskite obtained from ultraviolet photoelectron spectroscopy spectra. The schematic diagram of the band bending at graphene/perovskite interfaces is illustrated below; (**c**) Responsivity and (**d**) detectivity of the hybrid phototransistor as functions of light intensity at three selected drain voltages. Reproduced with permission for Figure 9a–d from 2017 Scientific Reports [77].

As an advanced step, the sequential vapor deposition method has been developed to grow ultra-flat MAPbI_3_ films on graphene, thereby forming a compact heterostructure for efficient light harvesting and exciton separation, as shown in Figure 9 [77]. This PD showed 1.73 × 10^7^ A/W *R* and 2 × 10^15^ Jones D*, several times larger than those of PD containing a MAPbI_3_ film prepared by conventional spin-coating method. Very recently, a thin layer of P3HT has been inserted between MAPbI_3−x_Cl_x_ perovskite and graphene as a hole transport layer for high-performance phototransistors [78]. In this study, the carrier diffusion length of the MAPbI_3−x_Cl_x_ perovskite was shown to be larger than that of the MAPbI­_3_ perovskite, resulting in more efficient transfer of the photo-generated holes to the graphene side. In addition, the separation of the photo-generated electrons and holes was more effective due to the presence of the P3HT layer, thereby reducing the recombination of the optical carriers, resulting in the transport of high-density electrons to the perovskite layer. The gain of this PD was close to 10^10^, at least one order of magnitude higher than those of other PDs without the P3HT layer [21,22,122]. 

Highly-crystalline perovskite nanostructures are advantageous for the photo-detection application because the recombination rate of the photo-generated carriers is much smaller in the bulk than in the thin film. Recently, the graphene/MAPbI_3_ NWs hybrid phototransistor was fabricated to show an *R* as large as 10^6^ A/W [79]. As another approach, the hybridization of graphene with CsPbBr_3−x_I_x_ nanocrystals (NCs) was done to fabricate phototransistors showing high *R* [80]. This PD also exhibited ultrahigh specific D* (2.4 × 10^16^ Jones) thanks to the extremely-high carrier mobility of the graphene, very promising for the application of the perovskite NCs in next-generation high-performance optoelectronics.

## 9. Graphene/Carbon Nanomaterials

Low-dimensional carbon materials such as fullerenes, carbon QDs, carbon nanotubes (CNTs), and graphene have attracted much interest over the past several decades due to their unique structural and physical properties [124,125,126]. Recently, various types of carbon nanomaterials have been integrated into hybrid structures including graphene/carbon nanomaterials, showing physical properties much better than those of the individual components [124,126]. 

### 9.1. Graphene/Zero-Dimensional Carbon

**Figure 10:**
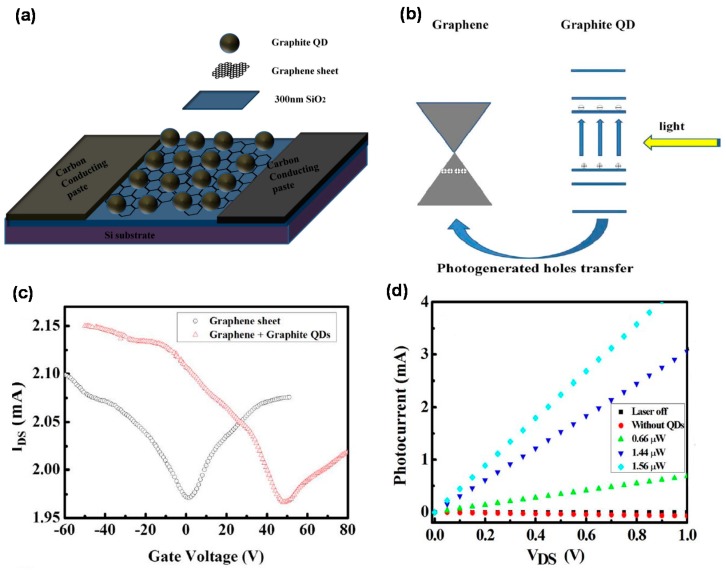
(**a**) Schematic of all carbon-based PD; (**b**) Under light illumination, the hole can transfer from graphite quantum dots to graphene sheets. The photo-generated electrons are trapped in graphite quantum dots; (**c**) Transfer characteristics of graphene transistors with and without the addition of graphite QDs on the graphene sheet; (**d**) Photocurrent of all carbon-based photodetectors for different optical powers as functions of drain-source voltage (*V_DS_*), showing a linear dependence on bias. The PC for the pure graphene device without graphite quantum dots is also shown here, which indicates a negligible effect. Reproduced with permission for Figure 10a–d from 2013 Scientific Reports [81].

Zero-dimensional carbon materials including graphite QDs, graphene QDs, and C60 were deposited on the graphene by spin coating or thermal evaporation methods to form hybrid structures for high-performance phototransistor applications [81,82,83,84,85]. In this structure, for example, graphite QDs act as light-harvesting material whilst the graphene layer is a conducting pathway for high-speed carriers, and carbon conductive paste was used as an electrode to complete the PD, as shown in Figure 10a [81]. While light is irradiated, the holes move to the graphene, shown in Figure 10b. In contrast, the electrons in the QDs act as an effective local gate to modulate the conductance of the graphene through capacitive coupling. The 300 nm SiO_2_ on Si was employed to investigate the properties of the intrinsic charge transfer between graphene and graphite QDs in the graphene FET, as shown in Figure 10a. Figure 10c shows the *I_DS_*-*V_g_* characteristics of the graphene FET before and after the deposition of the graphite QDs. The Dirac points are 1.6 V and 48 V before and after doping the graphite QDs, respectively. In other words, a higher *V_g_* is required to access the Dirac point of the graphite QDs/graphene FET because the net negative charge of the graphite QDs can attract more light-induced free holes in the graphene film. Such carrier transfer is known to enable more effective charge separation in graphite QDs and consequently suppress the electron-hole recombination [122,127]. Figure 10d shows PC as a function of drain-source voltage (*V_DS_*) under different optical powers, showing a linear dependence on bias. Here, the photoconduction gain is enhanced by the recycling of holes through the graphene channel within the lifetime of the trapped electrons. 

**Figure 11:**
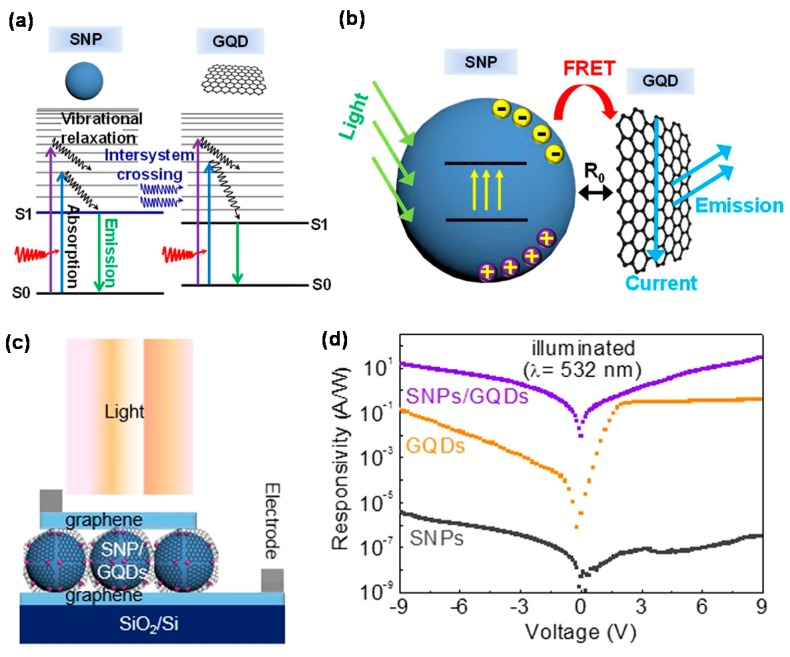
(**a**) Band structures of silica NPs (SNP) and graphene QD (GQD). Possible absorption/emission/transfer processes are described. Here, S0 and S1 indicate ground and lowest excited states, respectively. SNPs are highly absorptive, especially in the range of the emission spectrum of GQDs; (**b**) A schematic of a typical Förster resonance energy transfer (FRET) system composed of SNPs and GQDs as donors and acceptors, respectively. Photo-excited electron-hole pairs occupy mostly surface states of SNPs, thereby making it easy for the energy to be transferred to adjacent GQDs. The distance at which the FRET efficiency drops to 50% is defined as the Forster radius (*R*_0_), typically in the range of 1–10 nm. The use of one-atom-thick GQDs for acceptors can minimize *R*_0_ and maximize the contacting area of the acceptors and the donors; (**c**) Schematic of the PD structure composed of the FRET system sandwiched between single-layer graphene sheets; (**d**) Responsivities of SNPs, GQDs, and SNPs/GQDs hybrid as functions of bias voltage, excited at 532 nm. Reproduced with permission for Figure 11a–d from 2016 Scientific Reports [86].

Graphene was hybridized with graphene QDs on a rippled poly(dimethylsiloxane) substrate to fabricate stretchable PD [82], whose PC strongly depended on the external strain. The PC gradually decreased as the strain increased due to the reduction of light absorption resulting from multiple reflections of the photons in the ripple. The PD showed 2.8 × 10^3^ photoconductive gain in the UV range. Graphene/graphene QDs/graphene PD was also fabricated to form hybrid-type PD that operates over a wide spectral range from UV to NIR [84,85]. This PD showed ~0.5 A/W *R*, 10^11^ cm·Hz^1/2^/W D*, ~95 dB linear dynamic range (LDR), and 100 μs response time. Recently, PDs composed of silica NPs and graphene QDs as donors and acceptors were well operated based on Förster resonance energy transfer (FRET) mechanism, as shown in Figure 11a–c [86]. Under reverse bias at 532 nm, the *R* of the FRET system was more than three times greater than that of the PD containing only graphene QDs, as shown in Figure 11d. This performance improvement was interpreted by the network-like current paths formed by the graphene QDs on the silica NPs and easy transfer of the carriers generated from the silica NPs into the graphene QD due to their close attachment. The PD further exhibited asymmetric and nonlinear current–voltage characteristics, which was attributed to the tunneling of the charge carriers through the available density of graphene QDs between the metallic graphene layers.

### 9.2. Graphene/One-Dimensional Carbon 

CNTs can be metallic or semiconducting depending on their respective chiral vector [128]. Metallic CNTs are particularly useful as transparent conductors, whilst semiconducting CNTs are promising candidates as channel materials in FETs for electronic applications. CNTs are also very useful for new optoelectronic devices due to their inherent excellent optical properties such as direct bandgap nature, strong light absorption/emission, and high radiation/fluorescence lifetimes. Recently, there has been a report of a broadband phototransistor made of a graphene film on the ultrathin layer of single-wall CNTs [87]. In this device, the electrons move to the graphene channel and the holes are trapped in the CNTs. The trapped holes effectively modulate the channel conductance, leading to a continuous negative shift of the Dirac point voltage with increasing the illumination intensity. In addition, the built-in potential can be effectively tuned by adjusting the graphene Fermi level, thereby controlling PC and *R* under different back-gate voltages. The *R* was shown to be ~120 A/W and ~40 A/W at 650 and 1550 nm, respectively.

More recently, there has been a report on novel broadband graphene/CNT thin film photodiode [88], where the optical carriers near the interface are separated by built-in potential under illumination. The electrons and holes are then collected by each electrode, resulting in a PC in the external circuit. The *I_light_/I_dark_* ratio, *R*, and specific D* of the PD are 240, 0.21 A/W, and 4.87 × 10^10^ Jones, respectively, much larger than other CNTs-based PDs. The PD also exhibited a fast response rate of 68/78 μs rise/fall times and 5400 Hz 3dB bandwidth. 

## 10. Graphene/2D Layered Semiconductors

The van der Waals interaction between neighboring layers without dangling bonds in 2D materials allows for their integration with other materials, including graphene, to form a variety of planar or vertical functional heterostructures with fundamentally different properties. As a prototype geometry, 2D layered semiconductors such as InSe [89], WS_2__,_ [90], MoTe_2_ [91], and graphene were used as the light absorbing medium and both-side electrodes, respectively, to fabricate planar metal-semiconductor-metal-type phototransistors. Compared to metal electrodes, the graphene electrode has the advantage of tuning the Schottky barrier between the graphene and the 2D semiconductors by controlling the Fermi level of graphene. Therefore, the photoresponse characteristics can be adjusted by electrostatic gating or optical input control. An ideal ohmic contact between the graphene and the 2D layered semiconductor can be achieved by tuning the Schottky barrier.

Recently, the planar graphene/n-InSe/graphene van der Waals heterostructures showed high R of 4 × 10^3^ A·W^−1^ and fast rise/fall times of 1/10 ms [89]. The electrons move from graphene to n-InSe due to the higher work function of graphene, resulting in an accumulation layer at the graphene/n-InSe interface in equilibrium for a wide range of applied gate voltages. This study provides an innovative device architecture that enables access to fast electronic speed and high broadband spectral response. In another study, abnormal dependence of the *R* on incident light power was observed in graphene/WS_2_/graphene heterostructure device [90]. In this device, the *R* increases as the optical power increases at *V_g_* = 0 V (off state), considered to be due to an efficient photo-induced charge transfer process in which electrons reside in the graphene while the holes are located at WS_2_. On the other hand, at *V_g_* = 30 V (on state), the *R* decreases as the optical power increases. When on state in the phototransistor, the photo-gating effect can increase the Fermi level and reduce the contact resistance. As a result, the influence of illumination on the Fermi level of graphene is reduced, thereby causing different power dependence of *R*. 

In device structures dominated by photogating effects, 2D semiconductor serves as a medium for harvesting light, whereas graphene serves as a conduction channel for carrier transport and circulation. Recently, mechanically exfoliated graphene-MoS_2_ hybrid heterostructure phototransistors exhibited a high *R* of 5 × 10^8^ and 1 × 10^10^ A·W^−1^ at 130 K and room temperature, respectively [22]. Here, the photogenerated holes under illumination are trapped in MoS_2_ by local conditions, and the electrons are transferred to the graphene with the help of the gate electric field, resulting in high *R*. On the other hand, the trapped holes act as local gates, thereby causing a significant photogating effect on graphene through capacitive coupling. Eventually, the response time is slow because of the long life of the trapped holes. In another approach, a similar phototransistor was fabricated using CVD-induced graphene and MoS_2_ [92]. This hybrid heterostructure device showed a relatively low response of ~1.2 × 10^7^ A·W^−1^ due to the low carrier mobility of CVD-graphene and very slow response times of several-hundred seconds, but seemed to be better suited for practical applications by easy preparation in large areas. 

**Figure 12:**
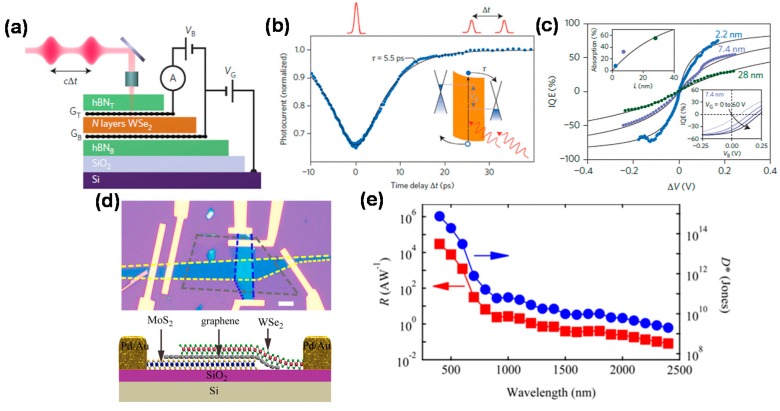
(**a**) Schematic illustration of the experimental time-resolved photocurrent set-up and cross-sectional view of the graphene/WSe_2_/graphene heterostructure; (**b**) Photocurrent as a function of time delay between two pulses; (**c**) IQE as a function of potential drop across theWSe_2_ layer *ΔV* for devices with channel length = 2.2, 7.4, and 28 nm. Reproduced with permission for Figure 12a–c from 2016 Nature Nanotechnology [95]; (**d**) Top: optical image of a device fabricated based on van der Waals-assembled MoS_2_-graphene-WSe_2_. Bottom: schematic side view of the heterostructure; (**e**) Responsivity (*R*) and specific detectivity (D*) of a typical device for wavelengths ranging from 400 to 2400 nm. The device was tested in ambient air at *V_DS_* = 1 V and *V_g_* = 0 V. Reproduced with permission for Figure 12d,e from 2016 Nano Letters [100].

Recently, graphene/transition metal dichalcogenide (TMD) heterostructures have also exhibited significant functionalities in the photodiode type. Carrier generation, separation, and transport processes were shown to be adjusted by easily modulating the Fermi level of graphene and the height of the Schottky barrier in graphene/TMD/graphene vertically-stacked heterostructures. For instance, graphene/WS_2_/graphene stack heterostructures were useful for efficient light–matter interactions, leading to improved photon absorption and electron-hole generation [93]. Scanning Photomicroscopy PC mapping showed that the PC generation occurred primarily in regions of heterogeneous structures with asymmetric potentials. In addition, *R* increases from ~10^−2^ to ~10^−1^ A·W^−1^ by integrating plasmonic metal nanostructures and heterostructures to improve the light absorption. It is worth noting that these results can be used for designing the dual-gate-type graphene/WS_2_/graphene heterostructure to control the polarity and amplitude of the PC by adjusting the direction and intensity of the built-in electric field [94]. A typical heterostructure of graphene/WSe_2_/graphene employing 2.2 nm trilayer WSe_2_, as shown in Figure 12a [95], exhibits fast response (5.5 ps) and high internal quantum efficiency (70%), as shown in Figure 12b,c. Further improvement of 7.3% in the external quantum efficiency was achieved by using optical waveguides and cavity or plasmon nanostructures in the TMD layer to improve light-matter interactions [129,130,131].

As another approach, relatively-low-*R* photodiodes were fabricated by integrating graphene with TMD stacked semiconductors such as MoS_2_, MoTe_2_, and WSe_2_ [96,97,98,99]. Here, the gate tunable mismatch of graphene and semiconductor Fermi levels allows tunable Schottky junctions, thereby showing adjustable rectification behaviors and photovoltaic response characteristics. Interestingly, the detection range of the devices can be extended enough to overcome band edge absorption limits of the semiconductor, useful for the internal photoemission in graphene. Especially, the graphene/MoS_2_ Schottky junction photodiode exhibited unique optical response over a wide spectral range from 400 to 1500 nm [96]. Maximum *R* values of 0.52 A·W^−1^ at 590 nm/1.26 A·W^−1^ at 1440 nm were obtained when operating in the energy gap excitation mode/when internal light emission dominated the PC generation in graphene, respectively. On the other hand, metal-insulator-semiconductor-like photodiodes with an insulator layer between graphene and MoS_2_ showed improved current rectification and higher current flow compared to 2D-based metal-semiconductor diodes and p-n junctions based on TMDs [97]. This was attributed to the formation of a multi-layered semiconductor, resulting in the carrier tunneling in the forward bias and the depressed carrier tunneling in the reverse bias. Figure 12d shows the vertical tuning of the WSe_2_/graphene/MoS_2_ gate tunable rectification behavior and the broadband response ranging from 400 to 2400 nm. [100]. The device exhibits high *R* and D* of up to 10^5^ A·W^−1^ and 10^15^ Jones in the visible range, respectively, but they sharply decrease to 10^−1^ A·W^−1^ and 10^9^ Jones at 2400 nm IR wavelength, as shown in Figure 12e. This unique response to different wavelengths can be understood as follows. In the visible light region where the incident photon energy is greater than the bandgap of the TMD, the incident light can be absorbed by all three materials (WSe_2_, graphene, and MoS_2_), resulting in a large number of photocarriers, contributing to significant increase of PC. However, when the IR light having energy smaller than the band gap of WSe_2_ is irradiated, only graphene can absorb light, resulting in very low PC. These results suggest that the van der Waals junctions based on 2D materials are very promising in future critical optoelectronic device applications.

## 11. Conclusions and Outlook

Graphene/semiconductor hybrid heterostructures showed their tremendous potential for PD applications. Particular attention was paid to several important issues related to device design, device performance, and processing technology to enhance figure-of-merit parameters of the PDs. By the optimization through various approaches, the PDs typically showed some new features that could open up the way to many conventional and advanced applications. The work function, surface resistance, and optical transparency of the graphene TCEs were successfully tuned by adjusting the number of layers, chemical doping, photo-induced doping, or electric field gating doping, which strongly affected the performance of the PDs. To meet the requirements for the practical applications of the PDs, some of the properties of the sensing materials should be optimized. Because the heterogeneity of the nanostructured materials can greatly affect the uniformity of the device performance, more precise control of their morphology, crystallinity, and orientation is highly needed. Some emerging methods such as plasmonic technology and integration of optical waveguides/micro cavities are also useful for improving the photo-material interactions, resulting in the increase of the light absorption. In particular, special efforts should be made for high response speed of the PDs by increasing the charge carrier mobility of the sensing materials or introducing a vertical electric field for the transfer and separation of photo-carriers. Broadband, spectrally selective, or flexible PDs should also be developed for specific applications. In addition to the improvements of the figure-of-merit parameters, long-term reliability and durability, environmentally-friendly and cost-effective machining technologies, large-scale production and consolidation are also important issues for various applications of the PDs in upcoming new situations.

## Figures and Tables

**Table 1:** Figure-of-merit parameters of semiconductor heterostructure photodetectors with graphene TCEs, reported until recently.

Device Structure	PC/DC	*R* (A·W^−1^)/Gain/EQE (%)	D* (cm·Hz^1/2^·W^−1^)	LDR (dB)	Recovery Time (ms)	Ref.
TPA-doped tri-layer graphene/Si	10^4^	EQE: 65 @ 550−800 nm	2.1 × 10^8^	-	3	[24]
Graphene/Si	-	*R*: 0.28 @ 1550 nm	-	-	-	[25]
Al_2_O_3_/graphene/Si	10^6^	EQE: 86.8 @ 200 nm	1.6 × 10^13^	127	5 × 10^−6^	[28]
Pt NPs/graphene/Si	-	EQE: 80 @ 790 nm	7.5 × 10^10^	-	7.8 × 10^−7^	[29]
MoO_3_/graphene/Si	-	EQE: 80 @ 550 nm	5.4 × 10^12^	-	-	[30]
Graphene/thin SiO_2_/Si	10^7^	EQE: 60 @ 650 nm	4.2 × 10^12^	90	7.5 × 10^−^^1^	[31]
Graphene/SiO_2_/Si	-	*R*: 3 @ 545 nm	3.5 × 10^12^	-	-	[32]
Graphene nanowalls/Si	-	EQE: 79.6 @ 810 nm	5.9 × 10^13^	105	4 × 10^−^^2^	[34]
Graphene/porous Si	-	EQE: 60 @ 500 nm	-	-	3 × 10^−^^3^	[35]
AuNPs/graphene/CH_3_-SiNW	10^6^	*R*: 1.5 @ 950 nm	2.5 × 10^14^	-	9.6 × 10^−^^2^	[36]
AuCl_3_-doped graphene/SQDs:SiO_2_	10^1^	EQE: 72 @ 600 nm	8.9 × 10^8^	73	1.6 × 10^−^^2^	[38]
TFSA-doped graphene/SQDs:SiO_2_	-	EQE: 84 @ 600 nm	1.1 × 10^10^	92	7 × 10^−^^3^	[39]
SQDs/graphene/Si	-	EQE: 80 @ 500 nm	7.4 × 10^9^	-	2.5 × 10^−5^	[40]
B-doped SQDs/graphene/Si	-	EQE: 10^12^ @ 532 nm	~10^13^	-	9 × 10^3^	[41]
Graphene/GaN	10^5^	*R*: 0.001 @ 350 nm	-	-	-	[42]
Graphene/GaN NWs	-	*R*: 0.25 @357 nm	-	-	-	[43]
MLG/GaAs	10^4^	*R*: 0.173 @ 850 nm	1.8 × 10^11^	-	1.2 × 10^−^^1^	[44]
Bilayer-graphene/Al_2_O_3_/GaAs	10^5^	*R*: 0.005 @ 850 nm	2.9 × 10^11^	-	4.8 × 10^−^^2^	[46]
ZnO QDs/graphene/SiO_2_/Si	-	*Gain*: 10^7^ @ 445 nm	-	-	10^3^	[49]
Graphene/ZnO Nanorod	-	*R* : 3 ×10^5^ @ 365 nm	-	-	6.6 × 10^4^	[50]
Fewlayer-graphene/ZnO NWs	-	*R*: 6.7 × 10^4^ @ 365 nm	10^11^	-	1.2 × 10^4^	[54]
ZnOQDs/graphene/SAM/SiO_2_/Si	10^7^	*R*: 3 × 10^9^ @ 335 nm	5.1 × 10^13^	-	2.3 × 10^3^	[55]
ZnO QDs core/Zn(Ac)shell/graphene/SiO_2_/Si	1.7	*R*: 10^9^ @ 330 nm	1 × 10^14^	-	8.5 × 10^4^	[56]
Graphene/ZnO NR arrays	10^2^	*R*: 113 @ 365 nm	-	-	3.6	[57]
Graphene/h-BN/ZnO	-	*R*: 1350 @ 365 nm	-	-	5.2 × 10^3^	[58]
Graphene/ZnO/Si	-	EQE: 80 @ 390 nm	3.9 × 10^13^	-	5.4 × 10^−^^1^	[61]
Graphene/ZnO	12.1	*R*: 3 × 10^4^ @ 365 nm	4.3 × 10^14^	-	2.2 × 10^4^	[62]
Graphene.ZnO NWs/graphene	-	*R*: 22.7 @ 370 nm	-	-	4.7 × 10^2^	[64]
ZrO quantum dots/graphene		*R*: 22 @ 266 nm			10^4^	[68]
MLG/Ga_2_O_3_		*R*: 39.3 @ 254 nm	5.9 × 10^13^		2.2 × 10^5^	[69]
Graphene/Ga_2_O_3_/graphene	10^3^	*R*: 10 @ 254 nm			9.6 × 10^2^	[70]
P3HT/graphene/PZT	-	*G*: 10^3^ @ 325 nm	-	-	1.6 × 10^3^	[71]
Pentacene/graphene/cPVP/Al_2_O_3_/AuNPs	-	*R*: 700 @ 520 nm	10^13^	-	-	[72]
C8-BTBT/graphene/SiO_2_/Si	-	*R*: 10^5^ @ 355 nm	-	-	8.3 × 10^2^	[73]
MAPbI_3_/graphene/SiO_2_/Si	-	*R*: 180 @ 520 nm	10^9^	-	5.4 × 10^2^	[74]
MAPbI_3_/graphene/Al_2_O_3_/PVP	-	*R*: 115 @ 515 nm	3 × 10^12^	-	5.3 × 10^3^	[75]
MAPbI_3_/graphene/AuNPs/SiO_2_/Si	-	*R*: 10^3^ @ 532 nm	-	-	1.7 × 10^4^	[76]
PMMA/MAPbI_3_/ODTS/SiO_2_/Si	-	*R*: 10^7^ @ 532 nm	2 × 10^15^		9 × 10^2^	[77]
MAPbI_3−x_Cl_x_/P3HT/SiO_2_/Si	-	*R*: 10^9^ @ 598 nm	-	-	10^4^	[78]
MAPbI_3_ NWs/graphene/SiO_2_/Si	-	*R*: 10^6^ @ 633 nm	-	-	7.5 × 10^4^	[79]
CsPbBr_3−x_I_x_/graphene/SiO_2_/Si	-	*R*: 10^9^ @ 405 nm	2 × 10^16^	-	3.6 × 10^3^	[80]
Graphite QDs/graphene/SiO_2_/Si	-	*R*: 10^7^ @ 325 nm	-	-	-	[81]
graphene QDs/graphene	-	*G*: 10^3^ @ 325 nm	-	-	-	[82]
Graphene QD/graphene/PZT	-	*R*: 10^9^ @ 325 nm	-	-	1.5 × 10^4^	[83]
Graphene/graphene QDs/graphene	10^3^	*R*: 0.5 @ 800 nm	10^11^	95	2 × 10^−3^	[84]
Graphene/Si NPs-graphene QDs/graphene	3	*R*: 0.31 @ 532 nm	-	-	-	[86]
Graphene/SWNT/SiO_2_/Si	-	*R*: 10^2^ @ 650 nm	-	-	10^−1^	[87]
Graphene/CNT/SiO_2_/Si	10^2^	*R*: 0.21 @ 980 nm	4.87 × 10^10^	60	7.8 × 10^−1^	[88]
InSe/graphene/SiO_2_/Si	-	*R*: 10^5^ @ 633 nm	-	-	10^1^	[89]
Graphene/WS_2_/graphene	54	*R*: 3.5 @ 532 nm	10^11^	-	-	[90]
Graphene/MoTe_2_/graphene	10^5^	*R*: 87 @ 473 nm	10^12^	-	2.3 × 10^1^	[91]
Graphene/MoS_2_/SiO_2_/Si	-	*R*: 107 @ 532 nm	-	-	-	[92]
Graphene/WS_2_/graphene	-	EQE: 30 @ 633 nm	-	-	-	[93]
Graphene/MoS_2_/graphene	-	EQE: 27 @ 514 nm	-	-	5 × 10^−^^2^	[94]
Graphene/WS_2_/graphene	-	EQE: 7.3 @ 759 nm	-	-	5.5 × 10^−9^	[95]
Graphene/MoS_2_/SiO_2_/Si	-	*R*: 1.26 @ 1440 nm	4.2 × 10^10^	-	-	[96]
MoS_2_/h-BN/Graphene	6.6	*R*: 0.3 @ 532 nm	-	-	1.1 × 10^4^	[97]
WS_2_/graphene/SiO_2_/Si	10^4^	*R*: 350 @ 532 nm	10^13^	-	3 × 10^−^^2^	[99]
WSe_2_/graphene/MoS_2_	-	*R*: 10^4^/10^−1^ @ 400/2400 nm	-	-	5 × 10^−^^2^/3 × 10^−^^2^	[100]
